# A novel high-low-high Schottky barrier based bidirectional tunnel field effect transistor

**DOI:** 10.1016/j.heliyon.2023.e13809

**Published:** 2023-02-17

**Authors:** Xiaoshi Jin, Shouqiang Zhang, Mengmeng Li, Xi Liu, Meng Li

**Affiliations:** School of Information Science and Engineering, Shenyang University of Technology, Shenyang, 110870, China

**Keywords:** Schottky barrier height, Schottky contact, TFET, Built-in potential

## Abstract

In this work, we proposed a novel High-Low-High Schottky barrier bidirectional tunnel field effect transistor (HLHSB-BTFET). Compared with previous technology which is named as High Schottky barrier BTFET (HSB-BTFET), the proposed HLHSB-BTFET requires only one gate electrode with independent power supply. More importantly, take an N type HLHSB-BTFET as an example, different from the previously proposed HSB-BTFET, due to that the effective potential of the central metal is increased with the increasing of drain to source voltage (V_ds_), built-in barrier heights maintain at the same value when the V_ds_ is increased. Therefore, there is no strong dependence between built-in barrier heights formed in the semiconductor region on the drain side and the V_ds_. Besides that low Schottky barrier formed on the interface between the conduction band of silicon regions on its both sides and the central metal (while high Schottky barrier formed between the valence band of silicon regions on its both sides and the central metal) have been designed for preventing the carriers in valence band from flowing into the central metal induced by thermionic emission effect. Thereafter, the proposed N type HLHSB-BTFET has a natural blocking effect on the carriers flowing in valence band, and this blocking effect is not significantly degraded with the increasing of V_ds_, which is a huge promotion from the previous technology. The comparison between the two technologies is carried out, which exactly agrees with the design assumptions.

## 1 Introduction

The development of integrated circuit technology depends on the reduction of device size, the improvement of device performance and the enrichment of device functions. For sub-30nm technology, short-channel effect of MOSFET with planar gate becomes serious. Therefore, multi-gate FET has been proposed and replaced planar MOSFET and significantly reduced the impact of short channel effect on the device subthreshold performance. The subthreshold swing (SS) of multi gate FET can be maintained at about 63mV/dec at room temperature, just like the planar MOSFET with micron channel length [1,2]. However, in order to further break through the bottleneck of the switch characteristics of MOSFET, tunnel field effect transistor (TFET) with band to band tunneling (BTBT) effect as the device conduction principle was proposed. Due to the more sensitive dependence between the tunnel current and the intensity of band bending, TFET achieves smaller SS than conventional MOSFET [[3], [4], [5]]. Both TFET and MOSFET are devices based on ion implantation and other doping processes. Since diffusion as the basic law of nature exists between heterogeneous mediums, and it is significantly accelerated in an environment with higher temperature, the manufacturing process used to form abrupt junction becomes complex for doping based devices in nanoscale. It requires highly difficult annealing process in millisecond. Expensive equipment for ion implantation is also required in accurate doping process. This significantly increases the necessary expenses for production. Comparing to the abrupt junction of MOSFET based on doping technology, Schottky Barrier MOSFET (SB-MOSFET) uses metal materials as the source and drain (S/D) regions of the device [[6], [7], [8]]. Due to that the enhancement of on state current can be achieved by adopting different alloy electrodes to form Schottky barrier with lower heights [[9], [10], [11], [12], [13]], the Schottky barrier height between the S/D electrodes and the conduction band of semiconductor region (φ_Bn_) is usually much lower than that between the S/D electrodes and the valence band (φ_Bp_) for n type SB-MOSFET [14]. Due to that the thermionic emission efficiency is decreased by the Schottky barrier and is decreased with the increasing of the Schottky barrier height, comparing to doping based MOSFET, It has been proved that SB-MOSFET can not achieve the same SS as doping based MOSFET [15]. More than that, the reverse leakage current of SB-MOSFET induced by BTBT is large [16]. An n type HSB-BTFET is proposed which adopt metallic junctions to form a higher Schottky barrier between source/drain contact and conduction band of silicon region [17]. Different from SB-MOSFET, it utilizes higher Schottky barrier to eliminate thermionic emission current as much as possible for off state and realize sharper abrupt metallic junctions to generate BTBT current as much as possible which is the generation mechanism of on state current. However, in order to block the formation of reversely biased leakage current, an assistant gate has to be designed in the central part and sets to work at a constant bias to form a potential barrier to prevent the electron-hole pairs generated by the BTBT phenomena from forming reversely biased leakage current. However, we found that if the drain to source voltage (V_ds_) is largely increased, the potential barrier generated by the assistant gate will be largely reduced and eventually lose the blocking effect to the electron hole pairs generated by the BTBT phenomena in reversely biased state, and finally lose the controllability of the leakage current. In this paper, we proposed a novel High-Low-High Schottky barrier bidirectional tunnel field effect transistor (HLHSB-BTFET). Comparing to HSB-BTFET, the proposed HLHSB-BTFET requires only one gate electrode with independent power supply. More importantly, take an N type HLHSB-BTFET as an example, different from the previously proposed HSB-BTFET, due to that the effective potential of the central metal is increased with the increasing of V_ds_, built-in barrier heights maintain at the same value when the V_ds_ is increased. Therefore, there is no strong dependence between built-in barrier heights formed in the semiconductor region on the drain side and the V_ds_. Besides that low Schottky barrier formed on the interface between the conduction band of silicon regions on its both sides and the central metal (while high Schottky barrier formed between the valence band of silicon regions on its both sides and the central metal) have been designed for preventing the carriers in valence band from flowing into the central metal induced by thermionic emission effect. Thereafter, the proposed N type HLHSB-BTFET has a natural blocking effect on the carriers flowing in valence band, and this blocking effect is not significantly degraded with the increasing of V_ds_, which is a huge promotion from the previous technology. The comparison between the two technologies is carried out, which exactly agrees with the design assumptions.

## 2 Device structure

**Fig. 1:**
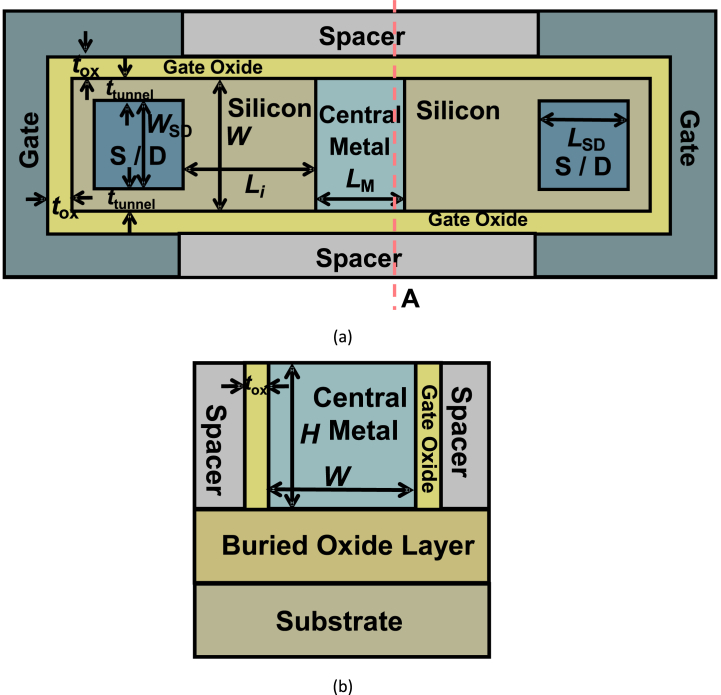
(a) Top view of HLHSB-BTFET, (b) cross section of HLHSB-BTFET along cutting line A in Fig. 1(a).

Fig. 1(a) shows a schematic top view of HLHSB-BTFET, Fig. 1(b) is a cross view of HLHSB-BTFET along cut line A in Fig. 1(a). The S/D regions are symmetrical and can change with each other. A significant difference between the proposed HLHSB-BTFET and the previous HSB-BTFET is that the central part of the device is replaced by a central metal instead of an assistant gate. The Schottky barriers are formed not only on the interface between source/drain electrode and silicon but also formed on the interface between central metal and silicon regions which are on each side of the central metal, respectively. However, it is worth noting that, taking n-type devices as an example, unlike the high Schottky barrier (barrier height larger than half of the energy band gap of silicon) formed between the source drain electrode and the conduction band of silicon regions, the Schottky barrier formed between the central metal and the conduction band of silicon regions are low Schottky barriers (barrier height smaller than half of the energy band gap of silicon). This makes the binding force of the central metal on electrons weaker than that of the semiconductors on both sides, resulting in some electrons in the central metal flowing to the semiconductors on both sides. Therefore, the potential of the part of semiconductors on both sides close to the central metal area will be higher than the part where the semiconductors on both sides are close to the source and drain electrodes. That is to say, a built-in potential difference is formed in the semiconductor areas on both sides. The formation of this potential difference helps to prevent holes on both sides of the source and drain from flowing to the central metal area, and also helps to prevent more electrons in the central metal from flowing to both sides of the source and drain. L_M_ is the length of the central metal. The other parts of the proposed HLHSB-BTFET are similar to the previously proposed HSB-BTFET. L_i_ represents the length of the undoped semiconductor region between the S/D contacts and the central metal. L_AG_ represents the length of the assistant gate. L_SD_ is the length of S/D contacts. t_ox_ represents the gate oxide thickness. t_tunnel_ represents the intrinsic tunnel layer thickness between the gate oxide and the S/D contacts. W_SD_ is the width of S/D contacts. W represents the width of the semiconductor region. H represents the height of the semiconductor region. The performance of HLHSB-BTFET has been analyzed and verified through simulation work by SILVACO [18]. Fermi-Dirac statistic model, SRH recombination model, auger recombination model, mobility models, band gap narrowing model, BTBT model, and Fowler-Nordheim dielectric tunneling model are all turn on.

## 3 Comparison and discussion

**Fig. 2:**
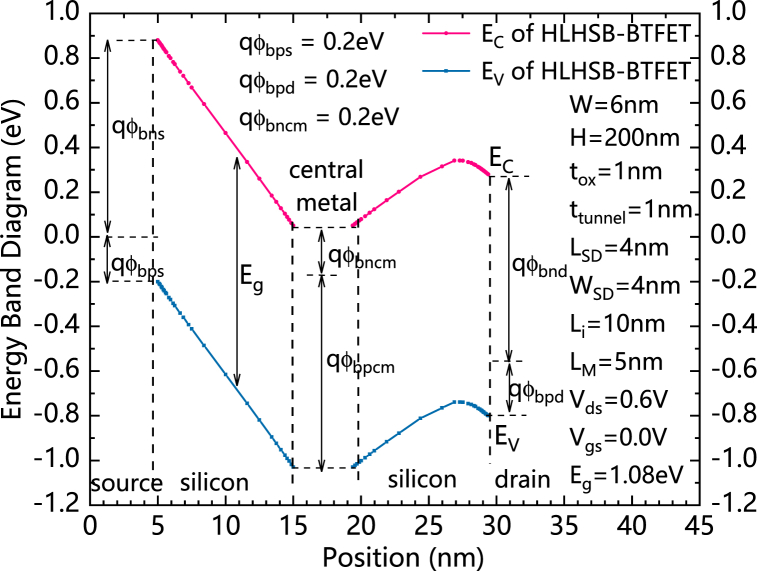
The energy band diagram of HLHSB-BTFET with V_ds_ equals to 0.6 V

Fig. 2 shows the energy band diagram of an n-type HLHSB-BTFET with V_ds_ equals to 0.6 V. qφ_Bns_ and qφ_Bps_ are the Schottky barrier heights for the conduction band and valence band of silicon on the source side, respectively. qφ_Bnd_ and qφ_Bpd_ are the Schottky barrier heights for the conduction band and valence band of silicon on the drain side, respectively. qφ_Bncm_ and qφ_Bpcm_ are the Schottky barrier heights for the conduction band and valence band of silicon in the central part, respectively. qφ_Bps_ and qφ_Bpd_ are set to equal to be 0.2eV. Therefore, qφ_Bns_ and qφ_Bnd_ are both set to be high Schottky barrier, which can strongly prevent thermionic emission current flow from source/drain electrode into the conduction band of silicon, a much lower qφ_Bncm_ is set to prevent the holes current from flowing through the central metal in the reversely biased state.

**Fig. 3:**
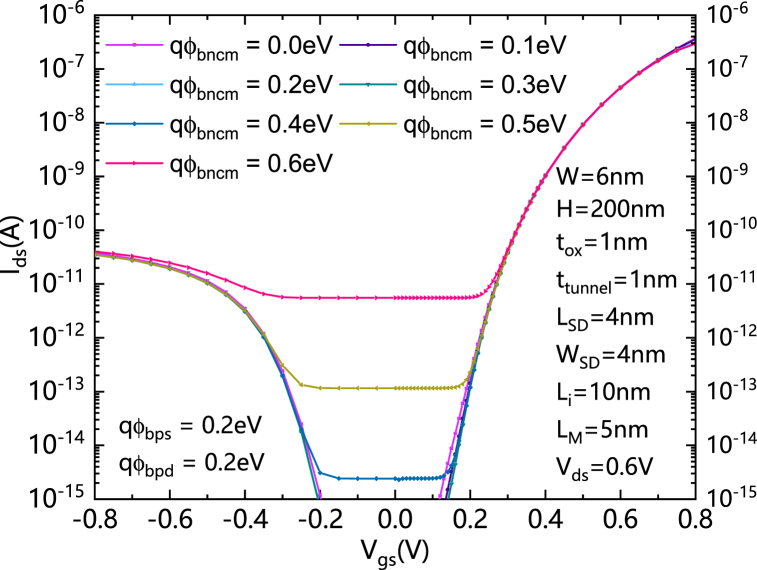
Comparisons of transfer characteristics of HLHSB-BTFET with different qφ_Bncm_ s.

Fig. 3 shows the comparisons of transfer characteristics of HLHSB-BTFET with different qφ_Bncm_ s. It can be clearly seen that when qφ_Bncm_ decreases, qφ_Bpcm_ increases at the same time. Therefore, the central metal gradually enhances the inhibition of the thermionic emission current generated in the valence band, so the static leakage hole current gradually decreases with the decreasing of qφ_Bncm_. Unlike the HSB-BTFET, which requires a constant assistant gate operating in the forward bias state, once qφ_Bncm_ is less than half the band gap width (about 0.5 eV), a good control effect on holes leakage current can be obtained.

**Fig. 4:**
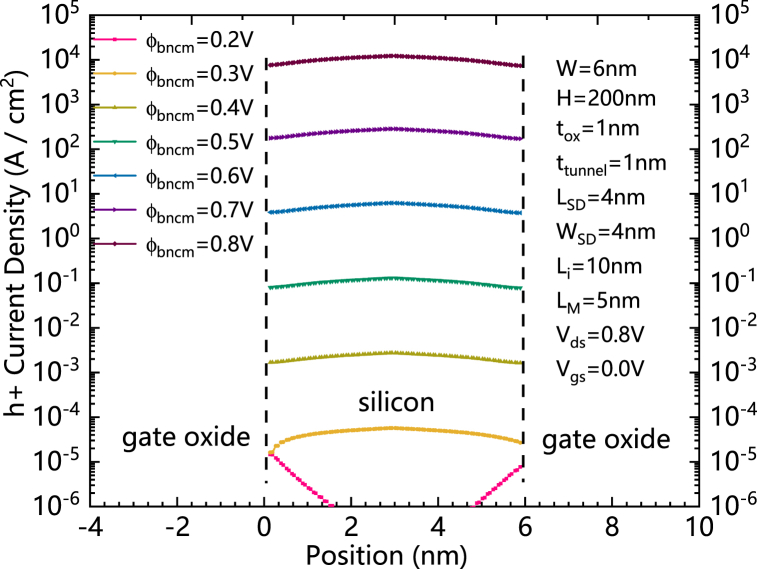
Comparisons of holes’current density of HLHSB-BTFET with different φ_Bncm_ s in silicon region. (For interpretation of the references to colour in this figure legend, the reader is referred to the Web version of this article.)

Fig. 4 shows the comparisons of holes current density of HLHSB-BTFET with different qφ_Bncm_ s in silicon region. As the qφ_Bncm_ is increasing, the qφ_Bpcm_ is decreasing at the same time. The Schottky barrier formed between the valence band of the silicon regions and the central metal is decreasing and the central metal gradually loses the ability to block the holes from flowing through the valence band. Therefore, the holes current density is increasing with the increasing of qφ_Bncm_.

**Fig. 5:**
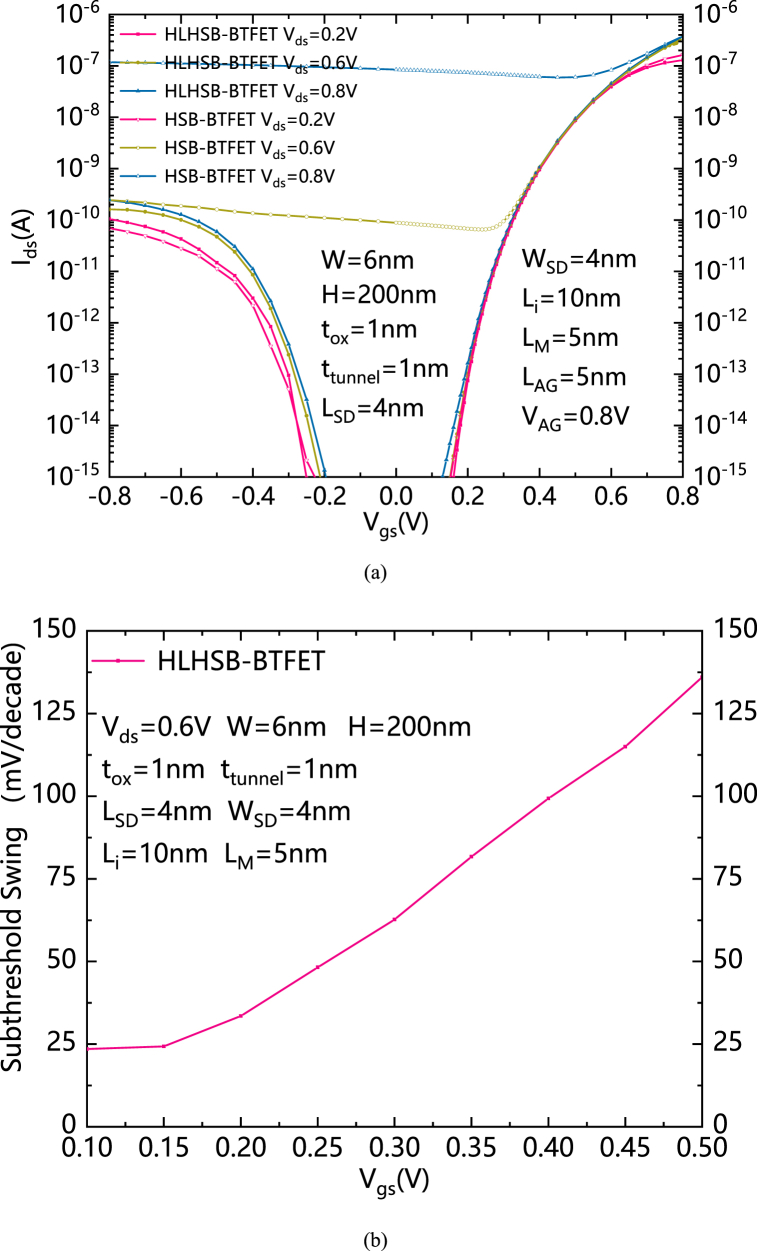
(a) Comparisons of transfer characteristics between HLHSB-BTFET and HSB-BTFET with different V_ds_s. (b) the relationship between SS and V_gs_ of HLHSB-BTFET.

Fig. 5(a) shows the comparisons of transfer characteristics between HLHSB-BTFET and HSB-BTFET with different V_ds_s. It can be clearly seen that when V_ds_ is low, HSB-BTFET and HLHSB-BTFET have relatively similar transfer characteristics. However, with the increase of V_ds_, HSB-BTFET begins to gradually lose its ability to control the holes leakage current. When V_ds_ rises to 0.8 V, the HSB-BTFET can hardly be turned off. On the contrary, the HLHSB-BTFET proposed in this paper is almost unaffected by the change of V_ds_. Fig. 5(b) shows the relationship between SS and V_gs_ of HLHSB-BTFET. Similar to other types of TFET, HLHSB-BTFET has obtained a lower subthreshold swing, which increases with the increase of gate voltage. In the entire subthreshold region, the average subthreshold swing of HLHSB-BTFET is 49mV/dec, which is lower than the subthreshold swing of MOSFET.

**Fig. 6:**
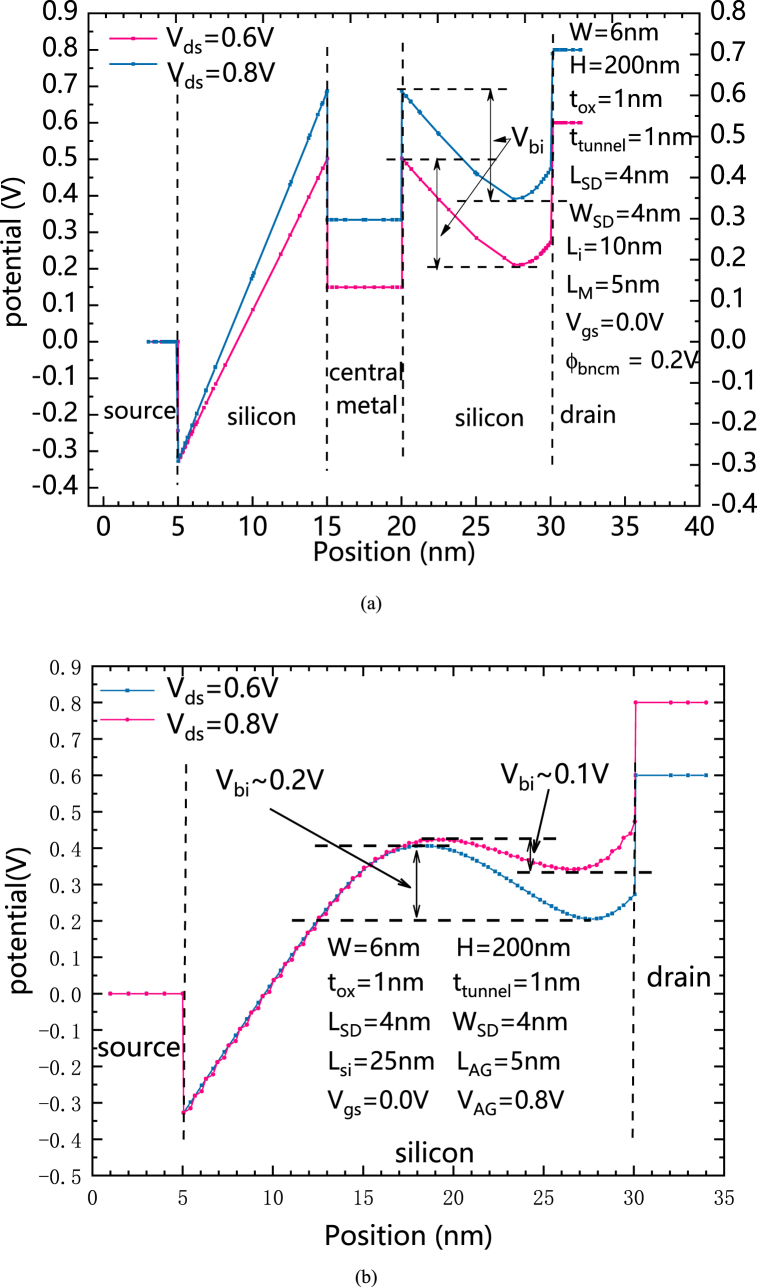
(a) Potential distribution of HLHSB-BTFET in silicon between the source electrode and the drain electrode with different V_ds_s, (b) Potential distribution of HSB-BTFET in silicon between the source electrode and the drain electrode with different V_ds_s. (For interpretation of the references to colour in this figure legend, the reader is referred to the Web version of this article.)

Fig. 6(a) shows the Potential distribution of HLHSB-BTFET from source to drain under different V_ds_s, and Fig. 6(b) shows Potential distribution of HSB-BTFET from source to drain under different V_ds_s. It can be clearly seen that, for HLHSB-BTFET, when V_ds_ increases, the potential of the central metal also increases, so the built-in potential difference formed in the semiconductor between the central metal and the drain electrode also does not change significantly. However, for HSB-BTFET, since the potential of the central silicon region is controlled by the assistant gate, when the assistant gate voltage is fixed to be a constant, The built-in potential difference inside the silicon near the drain side will decrease with the increase of V_ds_ difference, which will cause the assistant gate to lose its blocking effect on the holes flow from the drain side to the source side, thus generating a large amount of leakage current for higher V_ds_s.

**Fig. 7:**
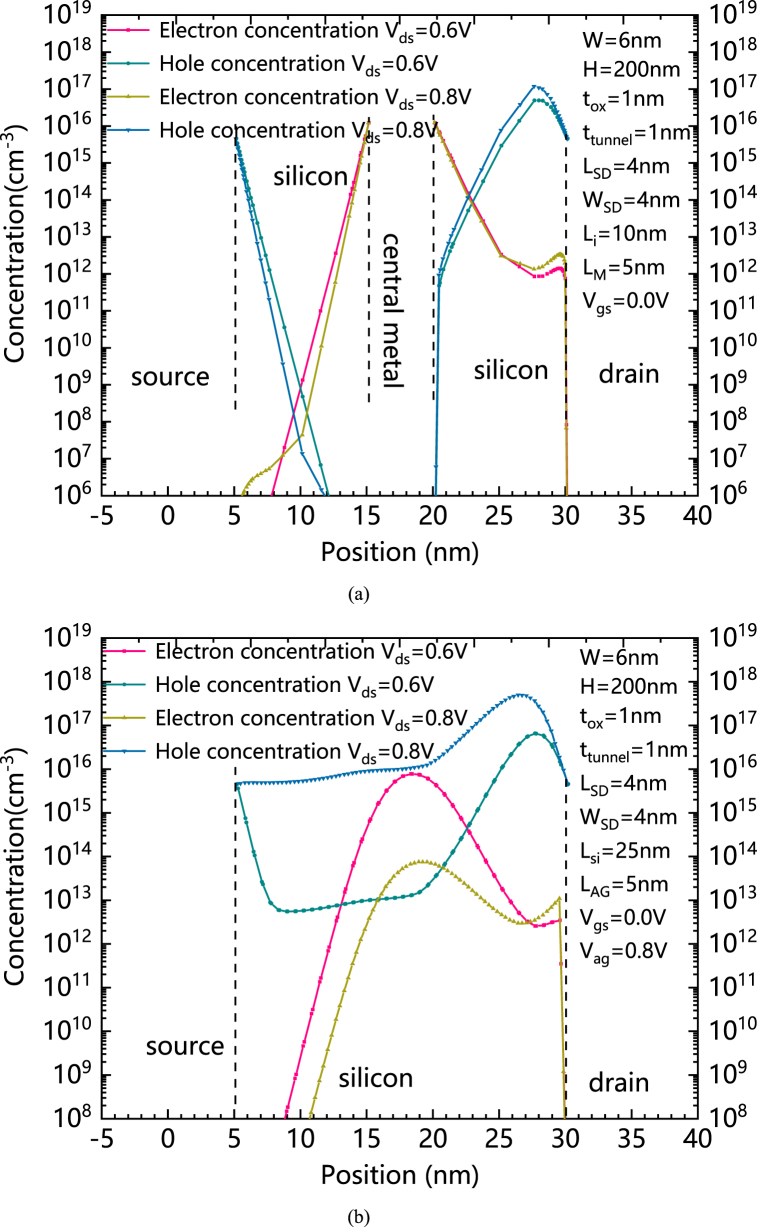
(a) Distributions of Electrons and holes' concentration of HLHSB-BTFET in silicon between the source electrode and the drain electrode with different V_ds_s, (b) Distributions of Electrons and holes' concentration of HSB-BTFET in silicon between the source electrode and the drain electrode with different V_ds_s. (For interpretation of the references to colour in this figure legend, the reader is referred to the Web version of this article.)

Fig. 7(a) shows the distributions of Electrons and holes’concentration of HLHSB-BTFET in silicon between the source electrode and the drain electrode with different V_ds_s, and Fig. 7(b) shows the distributions of Electrons and holes' concentration of HSB-BTFET in silicon between the source electrode and the drain electrode with different V_ds_s. In Fig. 7(b), it can be seen that a path of holes is formed in HSB-BTFET due to lose of hole blocking ability of the assistant gate for a higher V_ds._ On the contrary, for HLHSB-BTFET, the electron concentration near the central metal is always much higher than the hole concentration, so the P–N–P carrier distribution is formed in the direction from the source to the drain. Since the PN junction on the source side is always in the reverse bias state for a forward biased V_ds_, this also explains the physical reason for the low static leakage current of HLHSB-BTFET from another perspective.

**Fig. 8:**
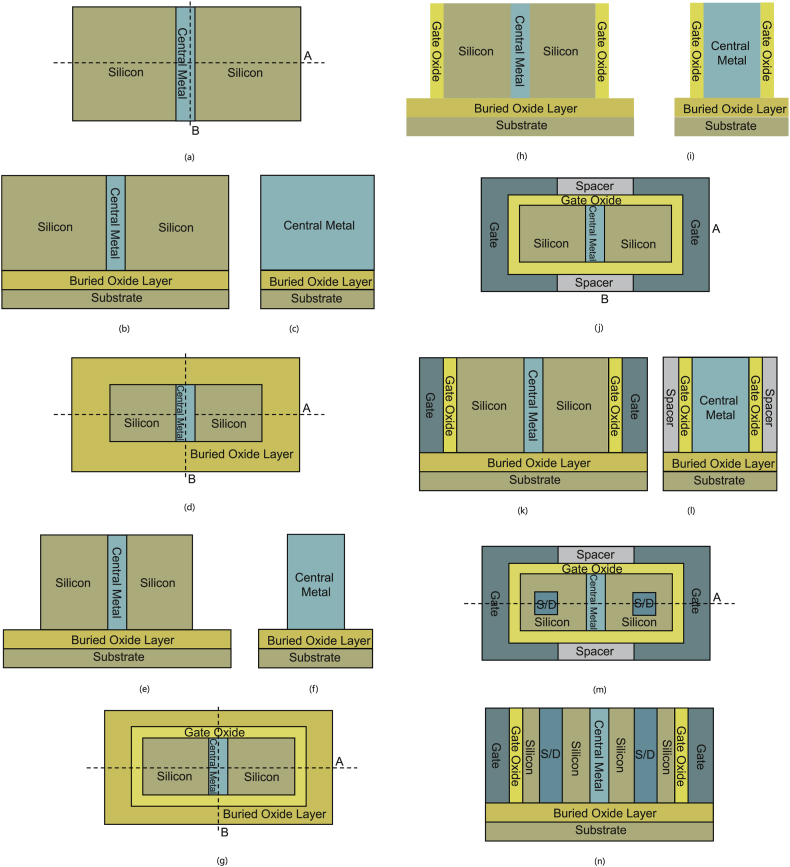
(a) Top view of step1, (b) cross view of Fig. 8(a) along cutline A, (c) cross view of Fig. 8(a) along cutline B, (d) top view of step 2, (e) cross view of Fig. 2, Fig. 8(d) along cutline A, (f) cross view of Fig. 8(d) along cutline B, (g) top view of step 3, (h) cross view of Fig. 8(g) along cutline A, (i) cross view of Fig. 8(g) along cutline B, (j) top view of step 4, (k) cross view of Fig. 8(j) along cutline A, (l) cross view of Fig. 8(j) along cutline B, (m) top view of step 5, (n) cross view of Fig. 8(m) along cutline A.

A brief fabrication flow of the proposed HLHSB-BTFET is shown from Fig. 8(a)–(n). As shown in Fig. 8(a)–(c), prepare a SOI wafer, the bottom of the SOI wafer is the silicon substrate. The top of the SOI wafer is the silicon film. The buried oxide layer is sandwiched between them. Remove the central area of the silicon film through the photolithography and etching process, and then deposit the first kind of metal material through the deposition process. After flattening the surface, the central metal is formed. As shown in Fig. 8(d)–(f), remove some areas around the silicon film and the central metal area to expose the buried oxide layer by photolithography and etching process. As shown in Fig. 8(g)–(i), through the deposition process, the insulating dielectric material used to form the grid oxide layer is deposited. After flattening the surface of the insulating dielectric material to expose the silicon film, the part of the area around the insulating dielectric material is removed to expose the buried oxide layer through the photolithography and etching process to form the gate oxide. As shown in Fig. 8(j)–(l), through the deposition process, deposit metal or poly silicon, flatten the surface to expose the silicon film, then remove part of the metal or poly silicon area above and below through the photolithography and etching process. Deposit insulating dielectric materials through the deposition process, flatten the surface again to expose the silicon film, thereafter, the gate electrode and spacer layer are formed through the above steps. As shown in Fig. 8(m) and (n), through photolithography and etching process, part of the area of the silicon film on the left and right sides is etched to expose the buried oxide layer. Then the second kind of metal material is deposited through the deposition process, and then the surface is flattened to expose the silicon film, and the metal source/drain interchangeable regions are formed through the above steps.

## 4 Conclusions

In this work, a novel High-Low-High Schottky barrier bidirectional tunnel field effect transistor (HLHSB-BTFET) is proposed. Compared with previous technology which is named as High Schottky barrier BTFET (HSB-BTFET), the proposed HLHSB-BTFET requires only one gate electrode with independent power supply. Due to that there is no strong dependence between built-in barrier heights formed in the semiconductor region on the drain side of the central metal and the V_ds_, besides low Schottky barrier heights formed between the central metal and the conduction band of silicon regions on its both sides have been designed for preventing the carriers in valence band from flowing into the central metal induced by thermionic emission effect, thereafter, the proposed N type HLHSB-BTFET has a natural blocking effect on the carriers flowing in valence band, and this blocking effect does not degrade significantly with the increasing of V_ds_, which is a huge promotion from the previous technology. The principle of the proposed HLHSB-BTFET has been explained through analysis on energy band theory. The influence of Schottky barrier heights has been quantitatively analyzed. Once qφ_Bncm_ is less than half the band gap width (about 0.5eV), a good control effect on holes leakage current can be obtained. And the holes current density in static state can be reduced to less than 10^−5^ A/cm^2^. The minimum SS is reduced to less than 25mV/dec, and the average SS in the entire subthreshold region is 49mV/dec. The physical mechanism that the proposed HLHSB-BTFET can better reduce static power consumption and reverse leakage hole current compared with HSB-BTFET is carefully analyzed by comparison of potential distributions and carrier concentration distributions. A brief fabrication flow of the proposed HLHSB-BTFET also has been given.

## Author contribution statement

Xiaoshi Jin; Xi Liu: Conceived and designed the experiments; Analyzed and interpreted the data; Contributed reagents, materials, analysis tools or data; Wrote the paper.

Shouqiang Zhang: Performed the experiments; Analyzed and interpreted the data; Contributed reagents, materials, analysis tools or data; Wrote the paper.

Mengmeng Li: Performed the experiments; Analyzed and interpreted the data; Contributed reagents, materials, analysis tools or data.

Meng Li: Analyzed and interpreted the data; Contributed reagents, materials, analysis tools or data.

## Funding statement

Meng Li was supported by the 10.13039/501100001809National Natural Science Foundation of China [62103288], 10.13039/501100003453Natural Science Foundation of Liaoning Province [2021-BS-151].

## Data availability statement

Data will be made available on request.

## Declaration of interest’s statement

The authors declare that they have no known competing financial interests or personal relationships that could have appeared to influence the work reported in this paper.
